# Coffee Consumption and Risk of Hypertension in Adults: Systematic Review and Meta-Analysis

**DOI:** 10.3390/nu15133060

**Published:** 2023-07-07

**Authors:** Fahimeh Haghighatdoost, Parisa Hajihashemi, Amanda Maria de Sousa Romeiro, Noushin Mohammadifard, Nizal Sarrafzadegan, Cesar de Oliveira, Erika Aparecida Silveira

**Affiliations:** 1 Isfahan Cardiovascular Research Center, Cardiovascular Research Institute, Isfahan University of Medical Sciences, Isfahan 8158388994, Iran; fhaghighatdoost@gmail.com (F.H.); nsarrafzadegan@gmail.com (N.S.); 2 Isfahan Gastroenterology and Hepatology Research Center, Isfahan University of Medical Sciences, Isfahan 8158388994, Iran; pari.hajihashemi1@gmail.com; 3 Postgraduate Program in Health Sciences, Faculty of Medicine, Federal University of Goiás, Goiânia 74605-050, Brazil; romeiroamanda@hotmail.com (A.M.d.S.R.); erikasil@terra.com.br (E.A.S.); 4 Hypertension Research Center, Cardiovascular Research Institute, Isfahan University of Medical Sciences, Isfahan 8158388994, Iran; 5 School of Population and Public Health, Faculty of Medicine, University of British Columbia, Vancouver, BC V6T 1Z3, Canada; 6 Department of Epidemiology & Public Health, Institute of Epidemiology & Health Care, University College London, London WC1E 6BT, UK

**Keywords:** hypertension, blood pressure, coffee, meta-analysis

## Abstract

Objectives: The association between coffee intake and hypertension (HTN) risk is controversial. Therefore, this systematic review and meta-analysis aimed at summarizing the current evidence on the association of coffee with hypertension risk in observational studies. Methods: PubMed/Medline and Web of Science were searched for observational studies up to February 2023. Observational studies which assessed the risk of HTN in the highest category of coffee consumption in comparison with the lowest intake were included in the current meta-analysis (registration number: CRD42022371494). The pooled effect of coffee on HTN was evaluated using a random-effects model. Results: Twenty-five studies i.e., thirteen cross-sectional studies and twelve cohorts were identified to be eligible. Combining 13 extracted effect sizes from cohort studies showed that higher coffee consumption was associated with 7% reduction in the risk of HTN (95% CI: 0.88, 0.97; *I*^2^: 22.3%), whereas combining 16 effect sizes from cross-sectional studies illustrated a greater reduction in HTN risk (RR = 0.79, 95% CI: 0.72, 0.87; *I*^2^ = 63.2%). These results varied by studies characteristics, such as the region of study, participants’ sex, study quality, and sample size. Conclusions: An inverse association was found between coffee consumption and hypertension risk in both cross-sectional and cohort studies. However, this association was dependent on studies characteristics. Further studies considering such factors are required to confirm the results of this study.

## 1. Introduction

Hypertension is directly associated with the risk of cardiovascular diseases [1]. The number of people aged 30 to 79 years with hypertension has increased from 648 million in 1990 to more than 1.2 billion people in 2019 [2], making it a serious public health concern, especially in low and middle-income countries [3]. Additionally, evidence suggests that high-quality diets are responsible for a 22% decline in the risk of cardiovascular disease [3,4], while the consumption of red and processed meats, high sodium intake, low potassium intake, obesity, alcohol consumption, as well as sugar-sweetened beverages are associated with an increase in the risk of hypertension [1,5].

Coffee is a beverage consumed daily by a large part of the world’s population [6]. Therefore, the effects of caffeine present in coffee have been studied in recent decades through several observational studies and clinical trials [7]. Caffeine can stimulate the production of adrenaline, which in turn has several effects on the cardiovascular system, such as increased blood pressure, endothelial dysfunction, inflammation, and decreased sensitivity to insulin, which may be associated with the risk of cardiovascular diseases [8]. 

Previous reviews have indicated that in healthy people, habitual coffee consumption is not associated with an increased risk of hypertension [9,10,11,12], especially when the amount of coffee consumed was greater than 3 cups per day compared with 1 cup per day [13]. Reviews of prospective cohort studies demonstrated that consumption of 1–3 cups of coffee per day may increase the risk of hypertension [14,15]. However, the results from these studies are controversial, due to variations in types of coffee and their composition, lifestyle, and study duration [14,15].

At present, there is not enough scientific evidence to confirm that consumption of coffee can act in the management of hypertension [16], especially in different populations from different regions of the world, such as America, Europe, and Asia. Given these disagreements in the existing literature, this is an updated systematic review and meta-analysis that included new published studies on the relationship between coffee and HTN risk aimed to summarizing the current evidence and exploring the potential sources of heterogeneity.

## 2. Methods

Search Strategy and Study Selection: This meta-analysis was designed, analyzed, and reported according to the Preferred Reporting Items for Systematic Reviews and Meta-Analyses (PRISMA) statement. A systematic search of the available publications was performed using the MEDLINE and Web of Science from inception (1952) to February 2023. Without any restriction on publication date and language, a search strategy applying the key terms of “(coffee OR caffeine) AND (hypertension OR “blood pressure”)” was conducted. No restriction was placed on the article language and publication date. A manual search of recent reviews and relevant original articles was performed for additional relevant studies. The study protocol was registered on the International Prospective Register of Systematic Reviews, PROSPERO (registration number: CRD42022371494). 

Study selection: Two independent reviewers (FH and PH) screened titles and abstracts for eligible articles. Disagreements were resolved by discussion with C.d.O. and E.S. Then, based on the full text of identified articles, their eligibility for inclusion was assessed according to our inclusion and exclusion criteria. Studies were included if they met the following criteria: (1) original studies on adult population (aged 18 or older), (2) an observational design (prospective cohort, case-control, or cross-sectional), (3) reporting the relative risk (RR) or hazard ratio (HR) or odds ratio (OR) with 95% confidence interval (CI), and (4) assessment of hypertension or elevated blood pressure in subjects with the highest vs. the lowest intake of coffee, irrespective of coffee types and their caffeine content. Studies were excluded if they were not original research or were in vitro or animal model, conducted on adolescents or children, examined gestational or ambulatory or coffee post injection blood pressure or hypertension risk, reported blood pressure mean, assessed blood pressure control over the time, and examined HTN risk for coffee polyphenols. Disagreements were resolved through discussion until agreement was reached. 

Data extraction and quality assessment: Using a pre-designed extraction form, two independent reviewers (PH and AM) extracted the following information: the author’s first name, country, year, study design, mean age or age range of participants, sample size, follow-up duration for longitudinal studies, instruments used to assess coffee intakes, the method and cut-off point used to HTN diagnosis, main findings (effect and 95% CI), and adjusted confounders. If the results of studies were reported in various groups, the information of all groups was extracted.

The quality of included studies in our meta-analysis was assessed using the New Castel Ottawa Scale (NOS) designed for observational studies. In general, this scale consisted of 3 main domains (selection, comparability, and outcome) and 8 questions in total for cohort studies. The minimum score for each domain is zero, while the maximum score varies between domains. The highest scores for participants’ selection, participants’ comparability, and assessment of outcome/exposure domains are 4, 2, and 3, respectively. The overall score ranges from zero to nine. In the adapted version for cross-sectional studies, consisting of 7 questions, the maximum score is 10. In the present study, the quality scores of ≥7 was considered methodologically high, and those with the score of 6 or fewer were considered low methodological quality [17,18]. 

### Statistical Analysis

Hypertension risk was reported either as relative risk (RR) or odds ratio (OR). Due to the high prevalence of HTN (32% in women and 34% in men) [2], ORs were converted into RRs [19]. To combine effect sizes, a random-effects model on the basis inverse-variance method, which incorporate between-study heterogeneity [20,21] was used. Potential between-studies heterogeneity was estimated using *I*^2^ values. Heterogeneity was considered substantial when *I*^2^ values were greater than 50% [21]. Heterogeneity sources were examined using sub-group analysis based on participants’ sex, study design, geographical region of studies, participants age (< vs. >50 years), sample size (< vs. >8000 in cohort studies and < vs. >3000 in cross-sectional studies), hypertension stage (stage I and II: SBP ≥ 130 mmHg and/or DBP ≥ 80 or 85 mmHg, and only stage II: SBP ≥ 140 mmHg and/or DBP ≥ 90 mmHg) [22], study quality score (≤7 vs. >7) [23], and follow-up duration for cohort studies. 

Publication bias was tested using visual inspection of a funnel plot, Egger’s test, and Begg’s tests [24,25]. Sensitivity analysis was performed to determine to which extent each individual study influences the pooled effect. All analyses were performed using the Stata 11.0 software. *p* values < 0.05 were regarded as significant.

## 3. Results

### 3.1. Search Results

**Figure 1:**
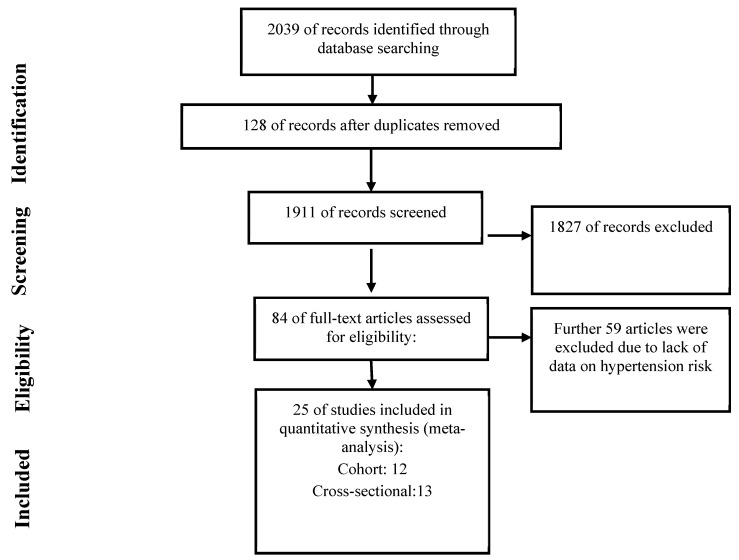
The flowchart of study selection.

The flowchart of study selection process is illustrated in Figure 1. Our search strategy identified 2039 articles. After removal of duplicate articles (*n* = 128), 1911 articles were screened for their titles and abstracts, of which 84 articles remained for further evaluation based on their full texts. Finally, 25 articles, published between 2002 and 2023, met our inclusion criteria and were included in the present meta-analysis [26,27,28,29,30,31,32,33,34,35,36,37,38,39,40,41,42,43,44,45,46,47,48,49,50]. Four of the twenty-five eligible articles were considered two separate studies or populations since they either consisted of two separate cohorts [45], or reported the results separately for men and women [48,49] or based on genes variants [40].

### 3.2. Characteristics of Studies Included in the Meta-Analysis

Table 1 and Table 2 show general characteristics of included studies in the current review. A total of 463,973 participants (321,978 from 12 cohort studies and 141,995 from 13 cross-sectional studies) were included in the meta-analysis. Overall, 11 studies were conducted in Europe [26,28,30,31,33,34,37,38,40,41,44], 9 studies in Asia [29,32,35,36,39,42,47,48,49], and 5 studies in the United States [27,43,45,46,50]. Twenty studies did not distinguish between sexes [26,27,28,29,31,32,33,34,35,36,37,38,39,41,42,43,44,47,48,49], two studies were conducted on men only [40,46], and three studies on women only [30,45,50]. Most studies evaluated the association between coffee and HTN risk, except for one which examined the associations for total antioxidants capacity of coffee [30]. One study assessed beverage consumption pattern identified by factor loading. This study was included since the pattern characterized by high consumption of unsweetened coffee and low consumption of sweetened coffee [35]. The evaluation of coffee consumption was quite heterogeneous among the included studies, with most of them classifying high coffee consumption above or equal to three cups a day [26,27,29,36,41,47], and low consumption was considered non-consumption or less than once a day or week. The volume of coffee per cup was evaluated in 8 studies [27,28,31,36,38,43,44,50], ranging from 50 to 237 mL. All studies were conducted on adults. A total of 8 studies determined blood pressure using the cut-off point of ≥130 and ≥80/85 mmHg for systolic and diastolic blood pressures [33,34,35,38,39,47,48,49], respectively, while others used the cut-off points of ≥140 and ≥90 mmHg, or taking anti-hypertension medicines of physician diagnosis. The majority of reported results suggested a null association between coffee and HTN risk (*n* = 18) [27,30,32,35,36,37,39,40,42,43,44,45,46,47,48,49,50], nine studies an inverse association [26,28,29,31,34,38,41,45,49], and one study suggested a positive direct link [33].

### 3.3. Results of Meta-Analysis

#### 3.3.1. Cohort Studies

**Figure 2:**
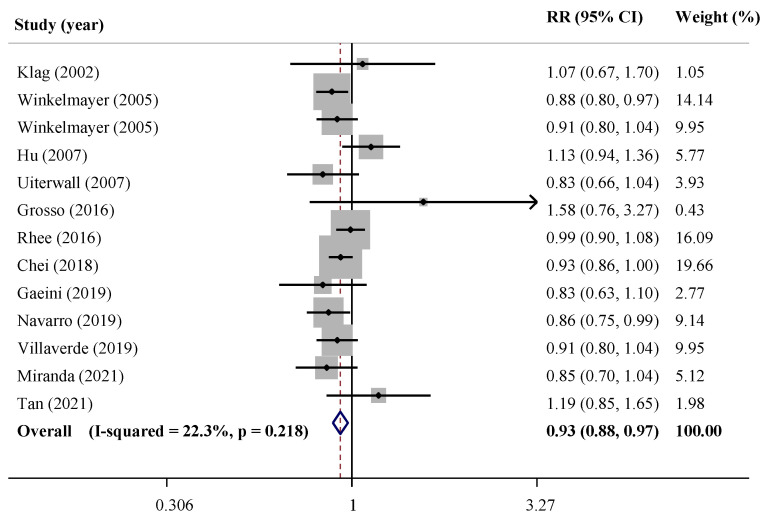
Forest plot of prospective cohort studies investigating the association of coffee with hypertension risk [27,30,31,32,36,37,43,44,45,46,47,50].

The pooled results of the meta-analysis revealed that individuals with the highest coffee consumption in comparison with the lowest intake had 7% lower risk for HTN (RR = 0.93, 95% CI: 0.88, 0.97; *I*^2^: 22.3%) (Figure 2). All selected cohort studies evaluated coffee consumption through a specific questionnaire. Findings from the sensitivity analysis demonstrated no change in the significance of the findings by excluding an individual study at the time. Despite a small asymmetry in the funnel plot, the Egger test (*p* = 0.449) and Begg test (*p* = 0.714) revealed no evidence of publication bias (Supplementary Figure S1). 

When studies were stratified based on the geographical region of the study, higher consumption of coffee compared with the lowest was associated with lower risk of HTN in studies which were conducted in the United States (RR = 0.92, 95% CI: 0.87, 0.97; *I*^2^ = 0.7%). However, it was not associated in Europe (RR = 0.97, 95% CI: 0.83, 1.13; *I*^2^ = 60.5%) and Asia (RR = 0.94, 95% CI: 0.83, 1.07; *I*^2^ = 25.0%). The results of subgroup analysis demonstrated an inverse association in the subgroups of studies with a follow-up duration of at least 10 years (RR = 0.91, 95% CI: 0.84, 0.99), sample size less than median (RR = 0.89, 95% CI: 0.81, 0.99) and cases more than median (RR = 0.93, 95% CI: 0.89, 0.97), and those which were low quality (RR = 0.88, 95% CI: 0.83, 0.94) or conducted in females only (RR = 0.93, 95% CI: 0.88, 0.98), while in the counterpart groups a null association was found. Subgroup analysis based on age showed similar results in both groups (Table 3).

#### 3.3.2. Cross-Sectional Studies

**Figure 3:**
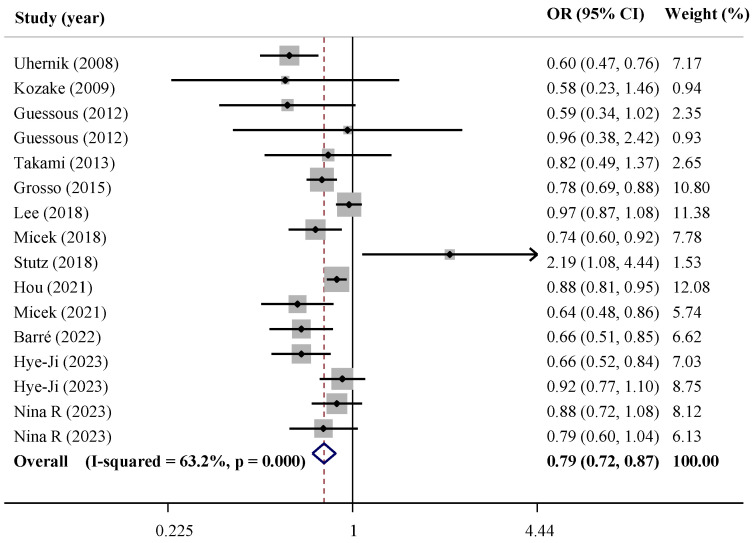
Forest plot of cross-sectional studies investigating the association of coffee with hypertension risk [26,28,29,33,34,35,38,39,40,41,42,48,49].

In cross-sectional studies, compared with the lowest amount of coffee consumption, the highest amount was associated with lower risk of HTN (OR = 0.79, 95% CI: 0.72, 0.87). Heterogeneity was considerably high between studies (*I*^2^: 63.2%) (Figure 3). Findings from the sensitivity analysis demonstrated no change in the significance of the findings by excluding an individual study at the time. No evidence of publication bias was observed (*p* for the Egger test = 0.198 and for the Begg test = 0.753). 

Subgroup analysis based on various variables did not change the association and could not eliminate the heterogeneity except for the subgroup of women (OR = 0.88, 95% CI: 0.75, 1.02; *I*^2^ = 0.0%), studies with smaller sample size (OR = 0.84, 95% CI: 0.67, 1.04; *I*^2^ = 47.3), and those which defined HTN using the cut-off point of 130/85 mmHg (OR = 0.88, 95% CI: 0.73, 1.06; *I*^2^ = 75.2%) (Table 3).

## 4. Discussion

The results of the present meta-analysis suggest, overall, a slight reduction in the risk of HTN following coffee consumption. This association was observed in both cohort and cross-sectional studies, and was much stronger in cross-sectional studies. However, this association was influenced by various factors in cohort and cross-sectional studies, such as geographical region, sex, sample size, and study quality.

There are several meta-analyses evaluating the relationship between coffee and HTN risk [12,51,52]. In the most recent meta-analysis, published in November 2022, 12 prospective cohort studies were included, and a null association was found [53]. However, this study has some methodological limitations. First, they included two studies which were conducted on hypertensive individuals. Second, they did not convert OR into RR in two studies despite the high prevalence of HTN. Third, the authors failed to include two relevant studies. In addition, another earlier meta-analysis on four prospective cohort studies conducted on general population [12] revealed a null association for habitual coffee consumption and HTN. However, we identified 6 further studies published after 2017 which were not included in their meta-analysis [27,30,31,32,36,47]. 

Overall, this meta-analysis suggested an inverse association between coffee consumption and HTN risk in both cross-sectional and cohort studies with a small heterogeneity in prospective studies. There is a large discrepancy between studies with regard to the association of coffee and HTN risk. While some studies suggested a favorable effect [26,28,29,31], others have reported a null or an adverse association [27,37,39,40]. The plausible mechanisms underpinning the inverse association between coffee and HTN risk might be attributable to high levels of antihypertensive nutrients (i.e., vitamin E, niacin, potassium and magnesium) and polyphenols in coffee [54]. These factors can modulate blood pressure through their antioxidant and anti-inflammatory properties, and effect on nitric oxide synthesis, lipid metabolism, and endothelial function [55,56]. Moreover, caffeine and chlorogenic acid (CGA) in coffee play a role in insulin homeostasis [57]. Insulin, in turn, causes sodium retention which activates symptomatic nervous system activity and the proliferation of vascular smooth muscle, leading to higher levels of blood pressure [58,59]. Other anti-hypertensive effects of CGA include its anti-inflammatory property [60], inhibitory angiotensin-converting enzyme activity [61], and vasodilation effect through increasing nitric-oxide bioavailability [62]. 

Our subgroup analyses indicated that the association between coffee and HTN risk differs by countries. Similar to an earlier meta-analysis, we found an in inverse association only in the USA, but not in Europe and Asia in cohort studies. This difference might be explained, at least to some extent, by the amount of coffee consumed. The inverse association between coffee and HTN risk is a non-linear association, and a coffee consumption less than 3 cups/day could not decrease HTN risk [12]. Based on the available evidence, the amount of coffee consumed in European and Asian countries was considerably lower than that of in the USA in 2016 [63]. In addition, the null association in Asian and European countries might be mediated by other variables, such as genetic factors [64], the method used to prepare the coffee, the kind of consumed coffee, and smoking [51,54]. It is also possible that variations in lifestyle and dietary habits in different parts of a continent lead to different association. For instance, Hu et al. [44] failed to find any significant association between coffee and HTN risk in Finland as a North European country, whereas Navarro et al. found an inverse association in Spain as a South European country. However, due to the small number of studies containing such information or located in a specific region, we could not further explore the reason behind this finding. 

The characteristics of studies, such as study quality, number of cases, and HTN definition could have affected the association. The results indicated an inverse association between coffee consumption and HTN risk in studies with a larger case number and those which used stage 1 cut-off point for HTN definition. A smaller case number cannot provide enough power to find the expected effect size, which is in concordance with our results as the confidence interval for studies with smaller case number was wider than those with more cases [65]. 

There was only one study which found a positive direct link between coffee consumption and HTN risk [33]. This study was conducted amongst patients with type 1 diabetes and approximately 50% of their study population consumed antihypertensive medications, which was considered a criterion for having HTN. This might restrict the generalizability of their findings per se. 

The main strengths of this meta-analysis are its large population from different countries and ethnicities with a wide age range, a comprehensive literature search to identify relevant articles, and subgroup analysis based on various confounders. Our findings were robust, and no evidence of publication bias was observed. In addition, most cohort studies were long enough to allow investigating incidence of hypertensive cases. We have also investigated a wide range of characteristics of studies which can influence the coffee-HTN association. The first and the main limitation of our study which should be considered for the future studies is the lack of adequate data for determining other plausible heterogeneity sources, such as sodium intake, smoking status, and the type of coffee and its preparing method. For instance, Grosso et al. showed that the association differs by the smoking status of participants. While in the whole population, they reported a null association with a wide confidence interval, in stratified analysis by smoking, they found an inverse association. They found that both non-smoker males and females who drank 3–4 cups coffee/d had lower risk of HTN development, whereas no significant association was found in smokers [37]. Furthermore, it is plausible that in countries which mainly tend to consume instant coffee, coffee consumption exhibits more favorable effects on health status due to its greater antioxidant activities in comparison with brewed coffee [66]. Second, between-studies inconsistency in terms of the adjusted confounders is another limitation which may explain between-studies heterogeneity, and should be considered. Third, estimating the amount of coffee consumption using different approaches, with their own specific measurement errors, may cause misclassification due to the combination of various errors. This, in turn, can affect the accuracy of findings. Fourth, heterogeneity was substantially high in the cross-sectional analysis and it could not be eliminated using subgroup analysis. Therefore, our findings should be interpreted cautiously. 

In conclusion, this meta-analysis showed an inverse association between coffee consumption and hypertension incidence, either cross-sectionally or prospectively. However, this association is dependent on the geographical region of the study, participants’ sex, the number of cases, and study quality. Future studies with an appropriate design which consider the effect of other confounders on HTN risk are warranted to confirm this result. 

## Figures and Tables

**Table 1:** General characteristics of included cohort studies in the meta-analysis.

First Author (Year)	Study Location/Setting	Sex/Age Range or Mean (Year)	Case/Total	Duration Follow-Up (Year)	Outcome Assessment/HTN Definition	Coffee Intake Assessment	Type of Coffee	Report of Coffee Intake	OR or RR or HR (95% CI)	Quality Assessment	Adjustments
Klag (2002) [46]	USA	M/26 years	281/1017	33 years	Self-report/≥160/≥95 mmHg	Questionnaire	Caffeinated	Coffee >5 vs. 0 cups/day	RR (95% CI) 1.07 (0.67–1.69)	6	Parental history of HTN, smoking, alcohol, physical activity, and BMI
Winkelmayer (2005) [45]	USA/NHS I and II	F/25–55 years	NHS I: 18,955/53,175 NHS II: 13,468/87,369	12 years NHS I: 1990–2002 and NSH II: 1991–2003	Self-reported diagnosis of HTN/≥140/≥90 mmHg	FFQ	Caffeinated and decaffeinated	Caffeinated coffee ≥6 vs. <1 cup/d	Caffeinated: RR (95% CI) NHS I: 0.88 (0.80–0.98) NSH II: 0.91 (0.80–1.04)	6	Age, BMI, alcohol, family history of HTN, oral contraceptive use (in Nurses’ Health Study II only), physical activity, and smoking
Hu (2007) [44]	Finland	M/F/25–64 years	2505/24,710	13.2 years	Self-reported initiation of antihypertensive drug treatment/Incidence of antihypertensive drug	FFQ/Questionnaire	NR	Coffee ≥8 vs. 0–1 cups/day	HR (95% CI) 1.13 (0.94–1.36)	7	Age, sex, study year, education, physical activity, smoking, alcohol, tea, frequency of vegetable, fruit, sausage, and bread consumption, BMI, history of diabetes, total cholesterol and baseline SBP
Uiterwall (2007) [43]	USA	M/F/Men: 40.7 ± 10.0 Women: 40.1 ± 10.3	956/5189	11 years	Measurement/≥140/≥90 mmHg	Questionnaire	Regular, decaffeinated or other	All type of coffees: >6 vs. 0 cups/day	Coffee: OR (95% CI) All: 0.83 (0.65–1.07) Men: 1.03 (0.72–1.46) Women: 0.67 (0.46–0.98)	8	Age, sex, BMI smoking, alcohol, tea, education level, occupational status, and total energy intake.
Grosso (2016) [37]	Poland/HAPIEE project	M/F/45–69 years	1735/2725	5 years	Measurement/>139/>89 mmHg	FFQ	NR but mostly caffeinated	Coffee >4 vs. <1 cups/day	OR (95% CI) All: 1.58 (0.85–3.64) Men: 2.42 (0.66–8.91) Women: 1.09 (0.36–3.33)	7	Age, sex, education, occupation, BMI, alcohol, smoking, physical activity, history of CVD, diabetes at baseline, cholesterol therapy at baseline, total energy intake, vitamin supplement use, oral contraceptives use, sodium and potassium intakes
Rhee (2016) [50]	USA/The Women’s Health Initiative Observational Study	F/50–79 years	5566/29,985	-	Measurement/≥140/≥90 mmHg	FFQ	Caffeinated and decaffainated	Caffeinated and decaffeinated: ≥4 vs. 0 cups/day	Caffeinated: HR (95% CI) 0.99 (0.90–1.08)	7	Age, baseline blood pressure, BMI, physical activity, hormone replacement therapy, alcohol consumption, smoking, total caloric intake, and intakes of sodium, magnesium, calcium, potassium, and phosphorus as time-varying covariates.
Chei (2018) [36]	Singapore/The Singapore Chinese Health Study	M/F/45–74 years	13,658/38,592	9.5 years	Self-report/-	FFQ	Caffeinated	Categorized Coffee ≥3 vs. 1 cups/day	HR (95% CI) 0.93 (0.86–1.00)	7	Age at recruitment (years), year of recruitment, sex, dialect group, BMI, education level, smoking, physical activity, sleep duration, and dietary intake of sodium, vegetables, fruits, and dairy products
Gaeini (2019) [32]	Iran/TLGS	M/F/≥19 years	291/1878	6 years	Measuring/≥140/≥90 mmHg or self-reported usage of blood pressure lowering medications	FFQ	NR	Coffee Drinkers vs. non-drinkers	HR (95% CI) 0.83 (0.63–1.10)	7	Sex, age, BMI, Triglyceride to HDL-C ratio, total energy intake
Navarro (2019) [31]	Spain/The SUN Project	M/F/35.7 ± 10.4	1750/13,369	9.1 years	Questionnaires/≥140/≥90 mmHg	FFQ	Regular and decaffeinated coffee	Caffeinated and decaffeinated coffee: ≥2 vs. 0 cups/day	HR (95% CI) 0.86 (0.75–0.99)	6	Age, BMI, alcohol, smoking and package-years of smoking, family history of HTN, sodium intake, whole and low fat dairy products consumption, sugar-sweetened beverages, non-sugared carbonated beverages, physical activity, adherence to Mediterranean diet, kind of personality), time spent watching TV and fried and fast-food consumption
Villaverde (2019) [30]	France/E3N	F/51.6 ± 6.2	9350/40,567	12.7 years	Questionnaire/-	Diet history questionnaire	NR	TAC (mmol/day) Quantile 5 vs. quantile 1	HR (95% CI) 0.91 (0.80; 1.04)	7	Age as the time scale, energy without alcohol, diabetes, treated hypercholesterolemia, education, family history of HTN, smoking, physical activity, BMI, Na, K, Mg, AGPIw3, alcohol
Miranda (2021) [27]	Brazil/ELSA	M/F/35–74 years	1285/8780	3.9 years	Measuring/≥140/≥90 mmHg And/or taking anti-hypertensive medications	FFQ	Caffeinated coffee	Coffee >3 vs. ≤1 cups/day	RR (95% CI) 0.85 (0.70–1.04)	6	Age, sex, race/skin color, educational attainment, household per capita income, BMI, physical activity level, smoking, alcohol, dietary intake of fruits, vegetables, sodium, potassium, saturated fat, added sugars, total energy intake, supplement use, fasting glucose, total cholesterol, and triglycerides
Tan (2021) [47]	Japan/HEXA	M/F/≥40 years	3897	5	Measurement/≥130/≥80 mmHg	FFQ	NR	Coffee >3 vs. 0 cups/day	RR (95% CI) 0.85 (0.64, 1.15)	7	Age, BMI, energy intake, educational level, current drinking status, current smoking status, and physical activity

**Table 2:** General characteristics of included cross-sectional studies in the meta-analysis.

First Author (year)	Study Location/Setting	Age Range/Mean Age (year)	Case/Total	Outcome Assessment	Coffee Intake Assessment	Type of Coffee	Report of Coffee Intake	OR or RR or HR (95% CI)	Quality Assessment	Adjustments
Kokaze (2009) [42]	Japan	M/53.8 ± 7.8 years	398	Measurement/≥140/≥90 mmHg	Questionnaire	NR	Coffee > 4 vs. ≤1 cups/day	OR ≤1: 1 2–3: 0.56 (0.34–1.01) >4: 0.58 (0.23–1.45)	6	Age, BMI, alcohol, smoking, serum total cholesterol level, serum HDL level, fasting plasma glucose level, serum uric acid level, and green tea.
Uhernik (2008) [41]	Croatia	M/F/>18 years	10,766	Measurement/≥140/≥90 mmHg	Questionnaire	NR	Coffee ≥3 vs. 0 cups/day	OR (95% CI) All: 0.6 (0.5–0.8) Men: 0.5 (0.3–0.7) Women: 1.1 (0.8–1.6)	6	-
Guessous (2012) [40]	Switzerland/The CoLaus study	M/35–75 years	6127/ Non-smokers: 4480 Smokers: 1647	Measurement/≥140/≥90 mmHg	Questionnaire	NR	Coffee >6 vs. 0 cups/day	OR (95% CI) Non-smokers 0.59 (0.34–1.02) Smokers: 0.96 (0.38–2.42)	8	Age, sex, BMI, contraceptive use, total cholesterol, triglycerides, diabetes, alcohol, CKD-EPI, CYP1A2 variants, menopause and *p*-value for interaction test.
Takami (2013) [39]	Japan/the Japan Multi-Institutional Collaborative Cohort (J-MICC) Study	M/F/35–70 years	172/554	Measurement/≥130/≥85 mmHg	Questionnaire	NR	Rarely <2, 3–4 and 5–6 cups/week 1–2, 3–4 and ≥5 cups/day	OR (95% CI) <1.5: 1 ≥1.5 and <3: 0.89 (0.55–1.45) ≥3: 0.82 (0.49–1.36)	8	Age, sex, total energy intake, physical activity, smoking and drinking habits
Grosso (2015) [38]	Poland/arm of the HAPPIE study	M/F/45–69 years	8821	Measurement/≥130/≥85 mmHg	FFQ	NR	Coffee ≥2 vs. <1 cups/day	OR (95% CI) All: 0.78 (0.69–0.88) Men: 0.88 (0.74–1.06) Women: 0.78 (0.65–0.95)	8	Sex, age, educational level, occupational level, physical activity, smoking, alcohol, total energy intake, and tea consumption
Lee (2018) [35]	Korea	M/F/19–64 years	15,713	Measurement/≥130/≥85 mmHg	24-h dietary recall	NR	Coffee pattern T3 vs. T1	OR (95% CI) 0.97 (0.87–1.09)	7	Sex, age, education level, income, smoking, physical activity, BMI (except for obesity and abdominal obesity), day of recalled intake; total daily energy intake.
Micek (2018) [34]	Poland	M/F/≥20 years	5164	Measurement/≥130/≥85 mmHg or treatment of previously diagnosed HTN	24-h dietary recall	NR	Coffee >400 vs. 0 g/day	OR (95% CI) 0.74 (0.60–0.92)	8	Sex, age, educational and occupational status, physical activity, smoking, alcohol, total energy intake, and tea consumption
Stutz (2018) [33]	Finland/the Finish Diabetic Nephropathy Study	M/F/46.7 ± 0.4	1040	Measurement/≥130/≥85 mmHg or use of hypertensive medication	FFQ	NR	Coffee ≥5 vs. <1 cup/d	OR (95% CI) 2.19 (1.08–4.44)	8	Age, sex, energy intake, alcohol, physical activity, and smoking
Hou (2021) [29]	Taiwan/Biobank database	M/F/30–70 years	3411/19,133	Questionnaire/-	Self-reports	NR	Coffee drinkers (at least thrice per week) vs. non-drinkers (habitually drank coffee less than three times per week)	OR (95% CI) 0.877(0.807–0.954)	5	-
Micek (2021) [28]	Italy/MEAL	M/F/≥18 years	2044	Measurement/≥140/≥90 mmHg Or medical history of taking anti-hypertensive medications	FFQ	NR	Categorized T3 (67.6 ± 39.5 mL/d) vs. T1 (47.6 ± 51.9 mL/d)	OR (95% CI) 0.64 (0.48–0.86)	9	Total energy intake, all beverages investigated, age, sex, educational status, smoking, physical activity level, adherence to the Mediterranean diet
Barré (2022) [26]	France	M/F/43 ± 14.81	4590	Self-report/Self-report or receiving treatment	Questionnaire	NR	Coffee ≥3 vs. 0 cups/day	OR (95% CI) 0.66 (0.51–0.85)	6	Age, sex, place of birth, living in a couple, tea, cannabis use, tobacco, alcohol, living in poverty
Hye-Ji An (2023) [49]	Korea/Korea National Health and Nutrition Examination Surveys	M/F/Men: 41.34 ± 0.15 Women: 42.39 ± 0.15	42,613 (17,311 men and 25,302 women)	Measurement/≥130/≥85 mmHg or medications	FFQ	NR	Coffee ≥1 time/d vs. <1 time/wk	OR (95% CI) Men: 0.66 (0.52, 0.84) Women: 0.92 (0.77, 1.11)	9	Age, the frequency of intake of tea, and carbonated beverages, daily nutritional intake (total and fat), income, education, smoking, alcohol drinking, walking, BMI, and menopausal status (only in women)
Nina R (2023) [48]	China	M/F/≥40 years	1719 (800 men, 919 women)	Measurement/≥130/≥85 mmHg	2-day, 24-h recall	NR	>1 serving/day vs. non-coffee drinkers	OR (95% CI) Men: 0.88 (0.72, 1.08) Women: 0.79 (0.60, 1.04)	9	BMI, education level, alcohol status, Physical activity

**Table 3:** Subgroup analyses for the association between coffee intake and hypertension risk.

	Number of Effects	Effect Size	95% Confidence Interval	*I*^2^ (%)	*p*-for between Subgroup Heterogeneity
Cohorts					
Region					0.924
US	6	0.92	0.87, 0.97	0.7	
Europe	4	0.97	0.83, 1.13	60.5	
Asia	3	0.94	0.83, 1.07	25.0	
Age					0.444
<50 years	7	0.91	0.84, 0.99	23.8	
>50 years	6	0.94	0.89, 1.00	28.5	
Sample size					0.244
<median (*n* = 20,000)	7	0.89	0.81, 0.99	12.6	
>median (*n* = 20,000)	6	0.94	0.84, 0.99	30.8	
Number of cases					0.967
<3000	8	0.94	0.84, 1.06	43.0	
>3000	5	0.93	0.89, 0.97	0.0	
Follow-up duration					0.506
<10 years	6	0.91	0.84, 0.99	22.3	
>10 years	7	0.94	0.88, 1.00	30.0	
Sex					0.833
Men	1	1.07	0.67, 1.70	-	
Women	4	0.93	0.88, 0.98	4.9	
Both	8	0.93	0.85, 1.02	41.3	
HTN stage					0.149
≥130 mmHg for SBP and/or 80 mmHg for DBP	1	1.19	0.85, 1.65	-	
≥140 mmHg for SBP and/or 90 mmHg for DBP	12	0.92	0.88, 0.97	17.7	
Study quality					0.056
Low	5	0.88	0.83, 0.94	0.0	
High	8	0.96	0.90, 1.03	34.2	
Cross-sectionals					
Region					<0.0001
Europe	8	0.72	0.62, 0.84	54.6	
Asia	8	0.87	0.81, 0.95	33.3	
Sample size					0.078
<median (*n* = 8000)	8	0.78	0.66, 0.91	48.5	
>median (*n* = 8000)	8	0.81	0.72, 0.90	70.9	
Sample size					0.733
<median (*n* = 3000)	7	0.84	0.67, 1.04	47.3	
>median (*n* = 3000)	9	0.78	0.70, 0.87	72.6	
Sex					0.423
Men	4	0.75	0.61, 0.93	31.4	
Women	2	0.88	0.75, 1.02	0.0	
Both	10	0.79	0.70, 0.89	73.4	
HTN stage					0.154
≥130 mmHg for SBP and/or 80 mmHg for DBP	5	0.88	0.73, 1.06	75.2	
≥140 mmHg for SBP and/or 90 mmHg for DBP	11	0.76	0.68, 0.85	55.7	
Study quality					0.637
Low	4	0.71	0.55, 0.91	77.0	
High	12	0.82	0.73, 0.91	59.9	

HTN: hypertension.

## Data Availability

Not applicable.

## Supplementary Materials

The following supporting information can be downloaded at: https://www.mdpi.com/article/10.3390/nu15133060/s1, Supplementary Figure S1: Funnel plots for cohort studies (A) and cross-sectional studies (B).
