# Supplementary figures and images for: Coffee Consumption and Risk of Hypertension in Adults: Systematic Review and Meta-Analysis
Source: Nutrients. 2023 Jul 7;15(13):3060. doi: 10.3390/nu15133060 (PMC10347253; doi:10.3390/nu15133060)

A

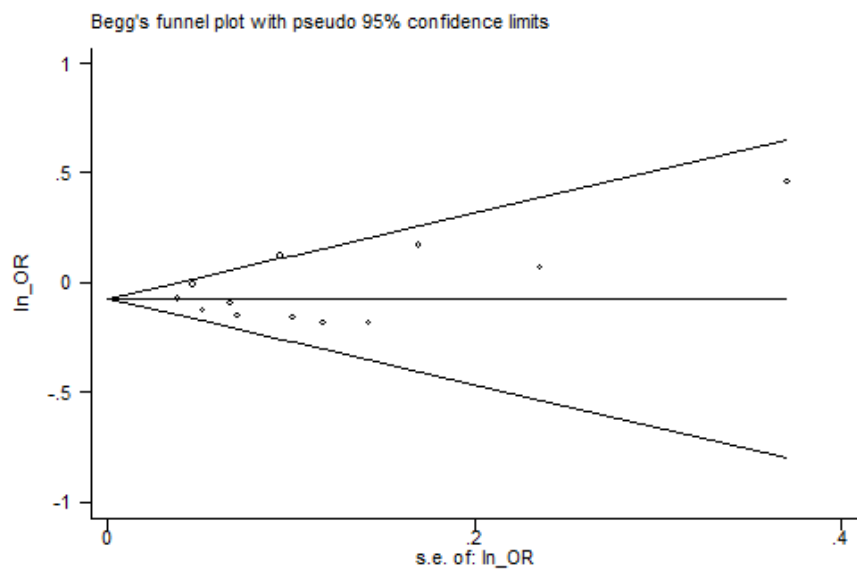

B

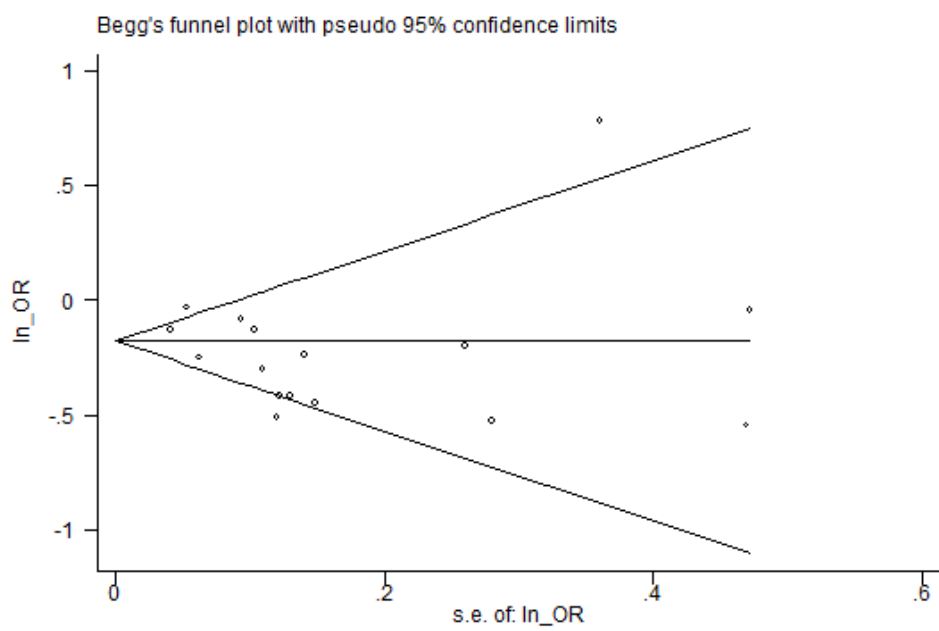

Supplementary Figure S1. Funnel plots for cohort studies (A) and cross-sectional studies (B).

Supplement: Supplementary file 1 [file nutrients-15-03060-s001.zip › nutrients-2464219-supplementary.pdf]
